# Perception and attitudes of street food vendors toward the healthiness of meals prepared and sold in Dodoma

**DOI:** 10.1002/fsn3.3374

**Published:** 2023-04-18

**Authors:** Zalia Omary Basheikh, Theresia Jumbe, Kissa Kulwa

**Affiliations:** ^1^ Department of Human Nutrition and Consumer Sciences Sokoine University of Agriculture Morogoro Tanzania

**Keywords:** attitudes, healthy meals, perceptions, street food vendors

## Abstract

Consumption of street meals among urban dwellers has become inevitable, especially in low‐ and middle‐income countries. It has been linked to higher incidence of dietary‐related diseases. Vendors' perceptions and attitudes toward the healthiness of meals can influence nutritional quality of the offered meals. Therefore, the study aimed to assess perceptions and attitudes of vendors toward healthy and unhealthy meals as well as the healthiness of meals they prepare and sell. A cross‐sectional study was carried out among 384 street food vendors. Face‐to‐face interviews were conducted using semistructured questionnaire. Pearson Chi‐square test and logistic regression analysis were used in comparing and testing for the association of perceptions and attitudes to vendors' characteristics. Street food vendors that took part in this study demonstrated good perceptions based on what they perceived to be healthy meals (58.33%) and positive attitudes (95.57%) toward preparation and provision of healthy meals. Perceptions were associated with sex (*p* = .007), education level (*p* = .002), and investment cost (*p* = .000). Results from logistic regression showed that better perceptions of healthy meals were associated with being female (OR = 2.46, *p*‐value < .031), having higher education (OR = 11.88, *p*‐value < .042), and vending experience of 1–5 years and more than 5 years (OR = 3.17, 2.95, *p*‐value < .019, .039, respectively) while having moderate investment cost showed significant lower chances of having better perceptions (OR = 0.33, *p*‐value < .001). Attitudes were associated with sex (*p* = .002), age (*p* = .002), marital status (*p* = .013), education (*p* = .009), and vending experience (*p* = .008). Female vendors, having 25 years of age and above, living with partners, with tertiary education, and having vending experience of more than 1 year had shown to have more positive attitudes toward healthy meals. Generally, street food vendors had good perceptions and attitudes toward healthy meals. This implies possible room for change and adoption of better ways of preparing meals. These findings could be used as a stepping stone in improving nutritional and healthy quality of street meals. Increased efforts are needed on the inclusion of nutritional aspects of healthy meals as they were merely considered by vendors. Future interventions on these vendors should focus more on male vendors, vendors with little vending experience, and little education as they had shown to have relatively poorer attitudes and perceptions compared to other groups.

## Graphical abstract

More than half of street food vendors had good perceptions of healthy meals based on how they defined a healthy meal and the meaning behind their definitions. Also, street food vendors had positive attitudes toward healthy meals. The determinants of vendors’ perceptions and attitudes toward healthy meals were found to be gender, education, and vending experience.

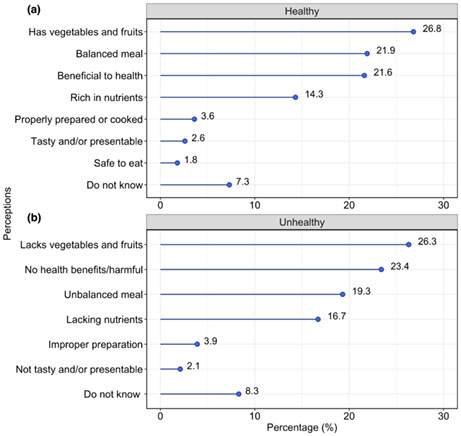

## 1 INTRODUCTION

Globally, noncommunicable diseases (NCDs) have become among the major public health challenges (Budreviciute et al., 2020; WHO, 2013). They are accountable for about 71% (41 million people) of deaths worldwide, 77% of which occur in low‐ and middle‐income countries (LMICs). In Tanzania, NCDs account for 27% of all deaths (MoHCDGEC, 2016). There are several key risk factors for NCDs such as the use of tobacco, excessive alcohol intake, physical inactivity, genetics, as well as unhealthy eating (WHO, 2021). Unhealthy diets are of particular concern, and these diets are the ones with inadequate amount of fruits and vegetables, excess amount of salt and sugar, and constitute high amount of carbs, saturated fats, and trans fats (World Heart Federation, 2011). These diets are associated with higher risk of developing NCDs such as heart diseases, stroke, cancer, and diabetes and their consumption has also risen significantly, especially in urban areas (GBD Diet Collaborators, 2017).

The nutritional and health status of individuals is affected by the quality of meals that are available in the places they live, with urban areas being no exception (Sawyer et al., 2021). The rapid urbanization and economic improvement in urban areas of LMICs have led to a dietary transition due to an increased demand for meat, sugar‐sweetened beverages, and processed foods (Popkin, 2015; Sousa et al., 2022). Studies in low‐ and middle‐income countries such as Tanzania, Uganda, and Nigeria have shown that there is an increase in consumption of meals lacking adequate fruits and vegetables, soft drinks, and sugar‐sweetened beverages, as well as high consumption of fast foods (Dolislager et al., 2022; Pallangyo et al., 2020). Nowadays, people increasingly spend more time away from homes due to income‐generating activities and therefore have limited time to prepare meals (Hawkes et al., 2017). This has led to the need for inexpensive, convenient, and ready‐to‐eat meals that are offered around streets.

Street food vendors have become vital in the provision of daily meals among urban dwellers, especially in LMICs, where approximately 70% of the population depends on them (Rosales Chavez et al., 2021). However, its consumption has been linked to the provision of unhealthy and nutritionally unbalanced meals (Mamiro et al., 2019), making it a public health concern. The nutritional composition of street meals is contributed by both homemade and processed foods which are characterized by energy‐dense foods per servings due to high carbohydrates and fat content in foods such as cakes, sweets, snacks, chips, pancakes, fried pastries, and main dishes. High sugary foods obtained from ultra‐processed foods such as soft drinks and also from traditional/homemade beverages due to additional sugar in the drinks (Albuquerque, Lança de Morais, et al., 2022; Bouafou et al., 2021; Steyn et al., 2014). High sodium per serving which is highly contributed by salt used in meal preparations. Sources of protein in street meals are majorly of animal origin, foods like stewed meat, and sandwiches, with less from vegetables and plant sources (Albuquerque et al., 2020). Also, street meals are characterized by low provision of fruits and vegetables on servings offered and sometimes are not provided at all (Albuquerque, Sousa, et al., 2022). The healthiness of street food meals is not only a result of its constituents, but it is also partly a result of the individual handlers who prepare and serve the food, i.e., street food vendors (Hossen et al., 2021). These vendors have not yet broadly embraced their roles in enhancing population and environmental health of the food system (Khongtong et al., 2014; Kolanowski et al., 2020). A report by WHO (2019c), has shown the nutritional inadequacy of street meals due to poor food preparations as conducted by vendors. Nutrition composition of these foods was found to be high in trans fatty acids and sodium due to the use of unhealthy ingredients during preparation such as too much salt and unhealthy fats.

While the increasing availability of street meals facilitates their consumption (Baidoe et al., 2020), the perception and attitudes of street food vendors on what it means in terms of the healthiness of the foods they prepare and sell are important. Most studies that have been conducted on street food vendors focused on their food safety and hygiene practices (Hassan & Fweja, 2020; Letuka et al., 2019; Ma et al., 2019; Madaki & Bavorova, 2019; Marutha & Chelule, 2020). Yet, street food vendors' perceptions and attitudes on the healthiness of foods being sold and provided are lacking. Their perceptions and attitudes on what healthy meals are, their composition, and their methods of preparation lead to meals being healthier have a great influence on the nutritional quality of the foods offered (Pinto et al., 2021).

Efforts on increasing street food vendors' perceptions and attitudes can be helpful in improving the nutritional quality of meals. People tend to adapt what are perceived to be good practices and avoid the ones that are perceived to be bad (Aggarwal et al., 2014; Gebremariam et al., 2018; Van Der Velde et al., 2019). In Tanzania and other LMICs, studies on perceptions and attitudes have been useful in improving the quality of street foods, especially on the hygienic and sanitary practices (Letuka et al., 2021; Mlay, 1993; Ncube et al., 2020). Similar approaches can be useful in improving nutritional quality of these foods. Thus, to promote healthy eating as a global goal in tackling NCDs, a better understanding of street food vendors' perception and attitudes on the healthiness of the foods they provide is needed. This will aid in planning better public health interventions that are based on pre‐existing perceptions and attitudes consequently enhancing the quality of diets being offered in streets. Therefore, this study investigated street food vendors' perceptions and attitudes on healthy and unhealthy meals as well as the healthiness of foods offered in Dodoma.

## 2 MATERIALS AND METHODS

### 2.1 Study setting

**FIGURE 1:**
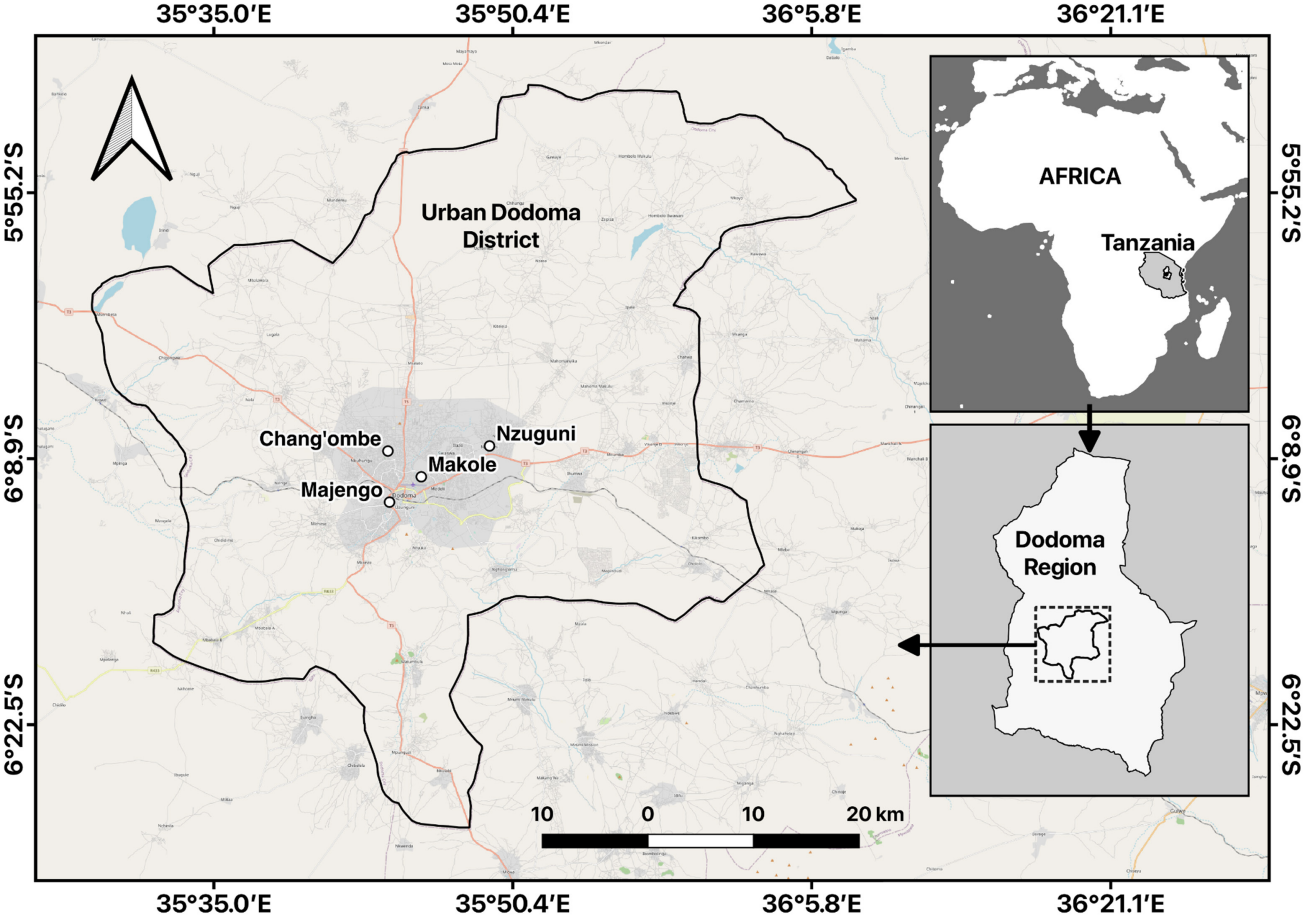
Map of the study area.

This research was carried out in Dodoma Urban district, located in the central part of Tanzania (Figure 1). It has a fast‐growing population of approximately 2,083,588 as reported in the latest census (URT, 2013). It was selected for this study because it serves as the government's administrative center and as a result, immigration of human labor is a common feature in this area. Within the district, four wards (Nzuguni, Makole, Chang'ombe, and Majengo) were selected due to the high concentration of street food vendors and convenience for data collection.

### 2.2 Study design and sampling procedure

A cross‐sectional study using quantitative and qualitative approaches was used in a population‐based sample between February and March 2022. The study included street food vendors based on the definition of street foods by FAO and WHO “ready‐to‐eat foods and beverages prepared and/or sold by vendors or hawkers, especially in the streets and other similar places” (FAO, 1988; WHO, 1996). Mobile street food vendors were excluded from the study. This study aimed at stationary vendors who permanently and on daily bases provide food to people around the markets and shops. Presumably, consumers can regularly and at any time obtain meals from these vendors. Therefore, their overall nutritional status is largely influenced by the consumption of these meals. A purposive sampling technique was employed to recruit the study participants. A total sampling size of 384 participants was calculated using Equation 1 (Kothari, 2004).

(1) $$n=\frac{z^{2}p\left(1-p\right)}{d^{2}}$$

where *n* is the desired sample size. The presumptions that served as the foundation for the estimation were as follows: *z* is the standard normal deviation set as 1.96 corresponding to a 95% confidence interval, *p* is the proportion of expected attitudes and perceptions of street food vendors set as 50% (since there were no similar studies in Tanzania), and *d* is the margin of error set at 5%.

Because the majority of vendors were concentrated around the markets, sampling procedure started with the identification of the major markets present in Dodoma Municipal. From the selected markets, all the vendors that were in and around the markets were approached for consent to participate in the study and those who agreed were included. Thus, convenience sampling was used in the study. But due to demolitions of some of the markets, vendors kept on shifting from the market to other places. Taking into consideration the intended sample size, street food vendors who were out of the intended radius were also included in the study.

### 2.3 Study tools and data collection

A semistructured questionnaire was developed for the Open Data Kit (ODK) (a simple, reliable, and effective mobile data collection tool) (Hartung et al., 2010). The questionnaire was pretested on 10 street food vendors who were not part of the study sample to verify the content validity and clarity and revised. The content of the tool used was validated by four experts on issues related to street food vendors validated the content of the questionnaire from Sokoine University of Agriculture (SUA) and the National Institute of Medical Research (NIMR). The questionnaire collected information on demographic data (sex, age, marital status, education level, investment cost, vending experience, and business certification), and perceptions and attitudes of street food vendors toward topics on healthy meals, composition, and preparatory methods. In both perceptions and attitudes, a total of 16 questions were asked, eight questions in each section. Perception questions were related to what vendors perceive to be healthy and unhealthy meals, composition of these meals, perceptions of various cooking methods, and their perceptions of unhealthy meals as related to noncommunicable diseases. Responses to the attitude questions were scored using a 5‐point Likert scale (5 = Strongly agree, 4 = Agree, 3 = Neutral, 2 = Disagree, 1 = Strongly disagree). The questions assessed vendors’ attitudes toward composition and outcomes of healthy and unhealthy meals, the need for street food vendors to provide healthy meals with the consideration of energy content in foods, fruits, and vegetables and the use of fats and oils.

### 2.4 Statistical analysis

Data were coded, entered, and analyzed using IBM SPSS statistics for Windows software, version 25 (IBM cop., Armonk, NY, USA). Categorical variables were summarized using frequencies (*n*) and percentages (%). Attitude variables toward healthy meals were categorized into positive (score ≥4), neutral (score 3–3.9), and negative attitudes (score <3) (Alhashim et al., 2022). Perception questions that were used for categorizations were based on three items, which are (1) their idea of a healthy meal, (2) which food groups a healthy meal comprises, and (3) what they consider to be good methods of cooking. For the idea of a healthy meal, if one mentioned balanced and nutritious, with health benefits, safe and properly prepared, they got one point for each. Five food groups used in the study were 1. Cereals, bananas, roots, and tubers, 2. Animal sources and legumes, 3. Fats/oils and honey, 4. Vegetables, and 5. Fruits whereby for each mention, 1 point was scored. Appropriate rating of whether a cooking method is healthy or unhealthy scored 1 point in each method. Cooking methods were 1. Boiling, 2. Steaming, 3. Frying, 4. Roasting, 5. Baking, and 6. Grilling. The total of all the points was 14 and it was used to obtain percentage score of each vendor. The total scores were then classified into two categories, that is, good and poor. A cut‐off point of 50% was used to categorize the “Good” (>50%) and “Poor” (<50%) levels of perception among the vendors (Nakwafila et al., 2022). These perception categories were used for comparison according to vendors' characteristics using Pearson Chi‐square test. Variables that were significantly associated at 5% level (*p*‐value < .05) from the Chi‐square test were inputted into a logistic regression analysis. The logistic regression analysis was used to determine the relative odds of having good perception of a healthy meal as opposed to poor perception, where the first level of each independent variable was controlled. Statistical significance for all analyses was set at *p* < .05.

### 2.5 Ethical considerations

The study was conducted according to research criteria on studies involving human participants and approved by the Tanzania National Institute of Medical Research (NIMR) and the Sokoine University of Agriculture. Other permissions were sought from Dodoma region, Dodoma Urban district, and from individual wards (Majengo, Makole, Nzuguni, and Chang'ombe). All the street sellers were informed of the study's goals, were given the opportunity to ask any questions they had, and provided informed verbal and written consent.

## 3 RESULTS

### 3.1 Characteristics of the vendors

Table 1 presents the sociodemographic characteristics of participating street food vendors in urban Dodoma. The majority of street food vendors who participated in this study (*n* = 384) were females (91.4%). More than ninety percent (96.4%) of the vendors were aged 25 years and above. Most had attained either a primary (64.8%) or secondary (27.1%) education and were married (83.6%). Most of the participating vendors reported having either an entrepreneurship or business license (81.5%). The majority of the vendors had an experience that ranged between 1 and 5 years (67.2%) with an investment cost between 50,000 and 100,000 TZS (59.1%).

**TABLE 1:** Sociodemographic characteristics of the street food vendors interviewed (*n* = 384).

Characteristic	Frequency (*n*)	Percent (%)
Sex		
Male	33	8.6
Female	351	91.4
Age (years)		
Young adults	295	76.8
Middle adults	85	22.2
Late adults	4	1.0
Marital status		
Single	50	13.0
Married/Cohabiting	321	83.6
Divorced/Widowed	13	3.4
Education levels		
None	23	6.0
Primary education	249	64.8
Secondary education	104	27.1
Tertiary education	8	2.1
Vending experience		
<1 year	23	6.0
1–5 years	258	67.2
>5 years	103	26.8
Entrepreneurship registration		
Yes	313	81.5
No	71	18.5
Investment cost (TZS)		
<50,000	72	18.8
50,000–100,000	227	59.1
>100,000	85	22.1

Abbreviation: TZS, Tanzanian Shillings.

### 3.2 Vendors' perceptions on healthy and unhealthy meal

**FIGURE 2:**
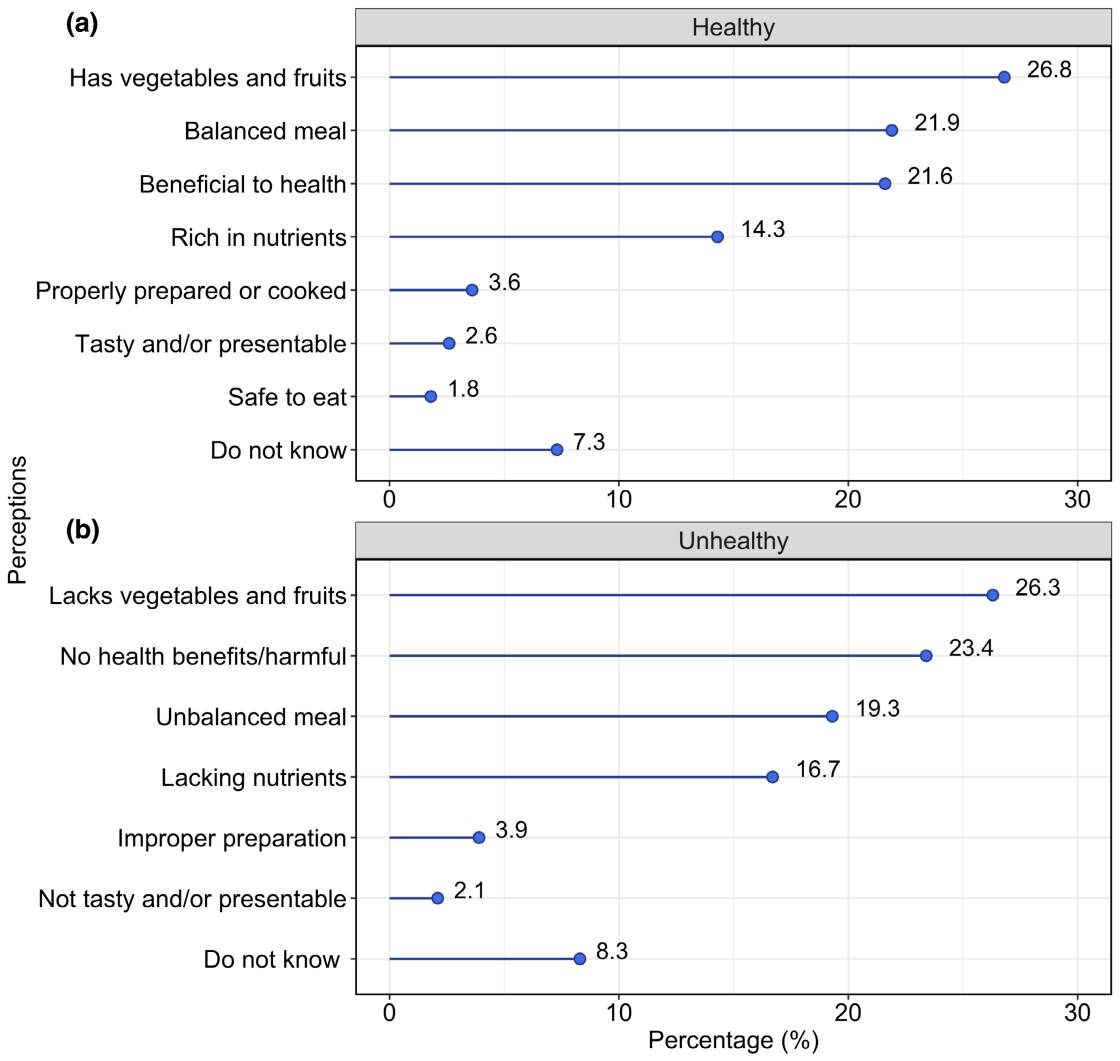
Vendors' perceptions about the meaning of (a) healthy and (b) unhealthy meals (*n* = 384).

According to FAO (2019), “A healthy diet is one which promotes growth and development, and prevents malnutrition”. Healthy meals are the ones that promote all dimensions of individuals' health, safety, and well‐being, comprising right proportions of the five groups prepared using more healthier methods that do not lead to additions of extra fats, salt, or free sugars from the preparation methods (Cena & Calder, 2020; Peeters, 2018; Shogo et al., 2021; World Heart Federation, 2011). Participants were requested to define what they perceive to be a healthy meal, and this was compared to the definition stated above. Many participating vendors demonstrated good perception levels (*n* = 224). Results in Figure 2a indicate the grouped ideas of what healthy diet was defined as by the vendors. Out of 384 vendors, 92.7% were able to describe what they perceived to be a healthy meal while 7.3% did not know. Vendors perceived healthy foods as those containing vegetables and fruits (26.8%), balanced, i.e., having more than two kinds of foods (21.9%), with health benefits (21.6%), rich in nutrients (14.3%), properly prepared/cooked (3.6%), tasty (2.6%), and safe to eat (1.8%). Regarding unhealthy meals, as shown in Figure 2b, vendors perceived unhealthy meals as those that lack fruits and vegetables (26.3%), are harmful to the body when consumed (23.4%), unbalanced (19.3%), lack enough nutrients (16.7%), not properly prepared/cooked (3.9%), not tasty and presentable (2.1%) while 8.3% did not share their perceptions on unhealthy meals.

A comparative analysis from the Chi‐square test showed a statistically significant association between the perception level of vendors and sex, education level, and investment cost (Table 2). A good perception level was significantly more frequent in female vendors, those who have received an education, and higher investment costs. No statistically significant differences regarding age, marital status, vending experience, and business registration were found.

**TABLE 2:** Perception classification of healthy meals by vendors’ characteristics.

Vendors characteristic	Good	Poor	*p*‐value
	*n* (%)	*n* (%)	
Sex			
Male	12 (36.4)	21 (63.6)	.007*
Female	212 (60.4)	139 (39.6)	
Age (years)			
Young adults	173 (58.6)	122 (41.4)	0.930
Middle adults	49 (57.6)	36 (42.4)	
Late adults	2 (50.0)	2 (50.0)	
Marital status			
Single	25 (50.0)	25 (50.0)	
Married/Cohabiting	192 (59.8)	129 (40.2)	0.401
Divorced/Widowed	7 (53.8)	6 (46.2)	
Education levels			
None	12 (52.2)	11 (47.8)	
Primary education	159 (63.9)	90 (36.1)	.002**
Secondary education	46 (44.2)	58 (55.8)	
Tertiary education	7 (87.5)	1 (12.5)	
Vending experience			
<1 year	8 (34.8)	15 (65.2)	
1–5 years	155 (60.1)	103 (39.9)	.061
>5 years	61 (59.2)	42 (40.8)	
Entrepreneurship registration			
Registered	181 (57.8)	132 (42.2)	.673
Not Registered	43 (60.6)	28 (39.4)	
Investment cost (TZS)			
<50,000	53 (73.6)	19 (26.4)	.000***
50,000–100,000	113 (49.8)	114 (50.2)	
>100,000	58(68.2)	27(31.8)	

*Note*: *p*‐value: Chi‐square test; *p < .05; **p < .01; ***p < .001.

Table 3 shows the odds ratio for having good perception levels of healthy meals, according to sociodemographic factors. Sex, education, investment cost, and vending experience were all found to have significant associations with the perception level of vendors. Female vendors were more likely to have good perception compared to the male (OR: 2.46, 95% CI: 1.10–5.76). Vendors having tertiary level education demonstrated increased odds of having a good perception (OR: 11.88, 95%: 1.46–260.23) compared to vendors having lower levels of education. Furthermore, vendors with an investment cost between TZS 50,000 and 100,000 had lower odds of having a good perception (OR: 0.33, 95%: 0.18–0.61) compared to vendors with investment costs below and above that range. Additionally, vendors that had an experience of more than 1 year also showed higher odds of having a good perception when compared to vendors who had less experience (OR: 3.17, 95% CI: 1.24–8.71; OR: 2.95, CI: 1.08–8.61).

**TABLE 3:** Odds Ratio (OR) of having good perception regarding healthy meals according to sociodemographic factors.

Variable	OR	95% CI	*p*‐value
Sex			
Male	Reference		
Female	2.46	1.10–5.76	.031*
Education			
None	Reference		
Primary education	1.41	0.54–3.64	.472
Secondary education	0.73	0.26–2.02	.546
Tertiary education	11.88	1.46–260.23	.042*
Investment cost (TZS)			
<50,000	Reference		
50,000–100,000	0.33	0.18–0.61	.001**
>100,000	0.91	0.46–1.76	.780
Vending experience			
<1 year	Reference		
1–5 years	3.17	1.24–8.71	.019*
>5 years	2.95	1.08–8.61	.039*

*Note*: *p*‐value: Logistic Regression; *p < .05; **p < .01.

Abbreviations: CI, Confidence Interval; OR, Odds Ratio; TZS, Tanzanian Shillings.

### 3.3 Attitudes toward healthy meals and their provision

**FIGURE 3:**
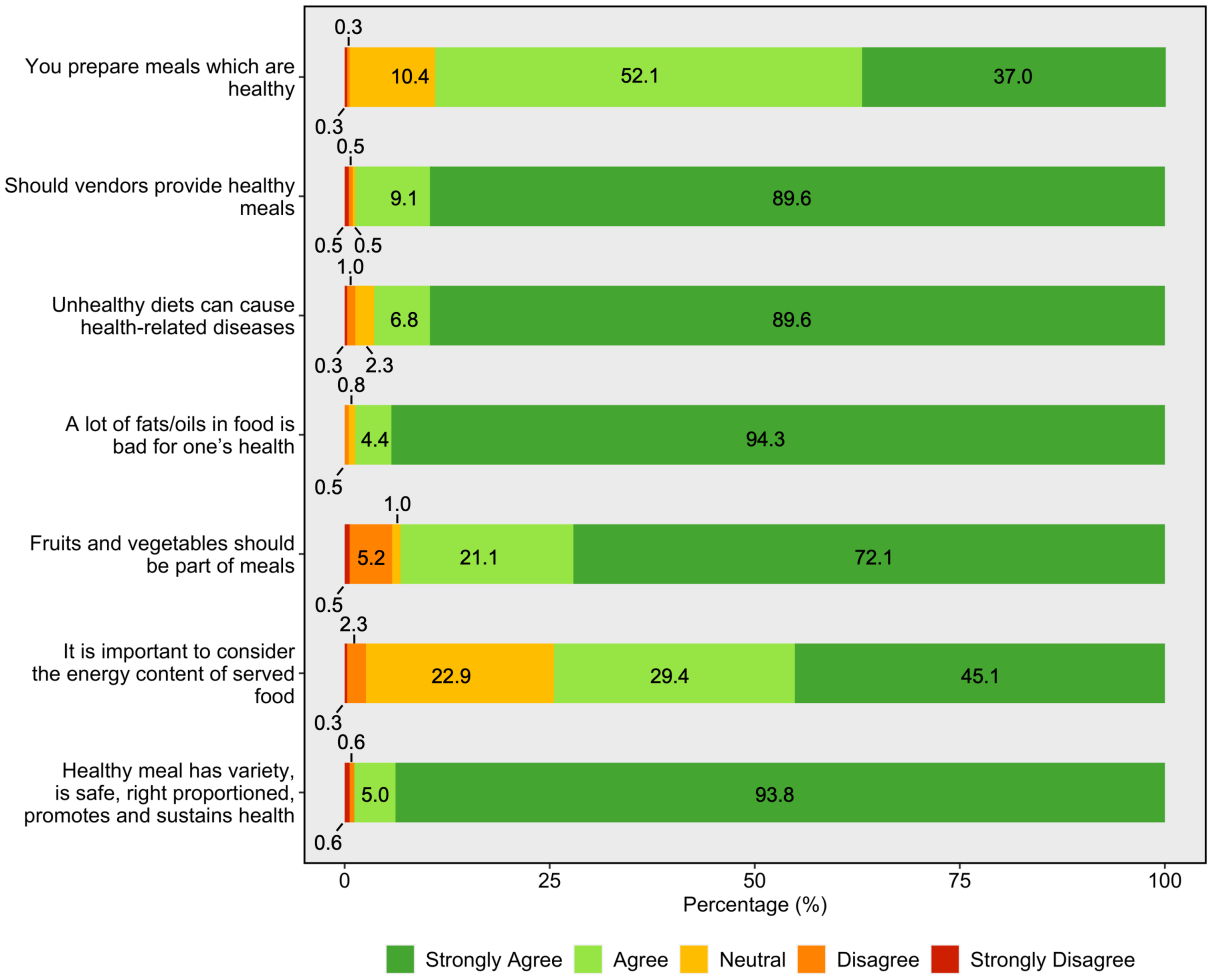
Distribution of street food vendors' attitudes toward healthy meals and healthiness of the meals they offer (*n* = 384).

Most street food vendors agreed or strongly agreed that a healthy meal has a variety of foods/ingredients or food groups, is safe, right proportioned, promotes and sustains health (99.0%), and that vendors should provide healthy meals to consumers (99.0%) (Figure 3). Most respondents agreed that unhealthy diets can cause health‐related diseases (96.0%), particularly that a lot of fats/oils in food are bad for one's health (99.0%). The majority of respondents also considered that they prepare healthy meals (89.0%). Around 93.0% of the respondents agreed that the inclusion of fruits and vegetables is a required part of meals served; however, a lower proportion (74.0%) agreed that it is important to consider the energy content of the meals being served.

There was a significant association between vendors' attitudes and the respondent's sex, age, marital status, education, and vending experience (Table 4). Participants who had a higher positive attitude were most frequently females (96.6%), older adults (96.2%), married (96.9%), received tertiary education (100.0%), and with vending experience of 1–5 years (96.5%).

**TABLE 4:** Distribution of street food vendors' attitudes toward healthy meals and healthiness of the meals they prepare and sell.

Vendors characteristic	Positive	Neutral	Negative	*p*‐value
	*n* (%)	*n* (%)	*n* (%)	
Sex				
Male	28 (84.8)	5 (15.2)	–	.004**
Female	339 (96.6)	11 (3.1)	1 (0.3)	
Age (years)				
Young adults	283 (95.9)	12 (4.1)	–	.436
Middle adults	80 (94.1)	4 (4.7)	1 (1.2)	
Late adults	4 (100.0)	–	–	
Marital status				
Single	45 (90)	4 (8.0)	1 (2.0)	
Married/Cohabiting	311 (96.9)	10 (3.1)	–	.090
Divorced/Widowed	11 (84.6)	2 (15.4)	–	
Education levels				
None	19 (82.6)	4 (17.4)	–	
Primary education	242 (97.2)	6 (2.4)	1 (0.4)	.035*
Secondary education	98 (94.2)	6 (5.8)	–	
Tertiary education	8 (100.0)	–	–	
Vending experience				
<1 year	19 (86.2)	4 (17.4)	–	.023*
1–5 years	249 (96.5)	8 (3.1)	1 (0.4)	
>5 years	99 (96.1)	4 (3.9)	–	
Entrepreneurship registration				
Registered	299 (95.5)	14 (4.5)	–	.910
Not registered	68 (95.8)	2 (2.8)	1 (1.4)	
Investment cost (TZS)				
<50,000	69 (95.8)	2 (2.8)	1 (1.4)	
50,000–100,000	217 (95.6)	10 (4.4)	–	.313
> 100,000	81 (95.3)	4 (4.7)	–	

*Note*: *p*‐value: Chi‐square test; *p < .05; **p < .01.

## 4 DISCUSSION

This study explored the perceptions and attitudes toward healthiness of meals offered by street food vendors in an urban area of Tanzania. Participants were predominantly females, similar to other reported studies in Africa where cooking is viewed as a females' role and women are also the breadwinners in their households (Letuka et al., 2021; Marutha & Chelule, 2020). Major findings of this study were as follows: (1) more than half of the vendors had good perceptions of healthy meals based on how they defined a healthy meal and the meaning behind their definitions, (2) vendors had positive attitudes toward healthy meals, and (3) gender, education, and vending experience were determinants of perceptions and attitudes toward healthy meals.

### 4.1 Perception of street food vendors

The perceptions of healthy meals that were found in this study were related to the improvement of health of the consumers as an outcome of a particular meal consumption. Vendors perceived that preparation of balanced meals with the inclusion of fruits and vegetables results in healthy meals. These results could be an outcome of increased awareness on the importance of fruits and vegetables as well as promoting consumption of these foods in the general population. This was similarly found in Ghana among food retailers (Nanema et al., 2022). Their general perception of healthy meals is, thus, closely related to what healthy meals have been described as by the World Health Organization (WHO, 2019b) where a healthy meal is considered to be the one that can protect one from malnutrition and dietary‐related diseases. However, it was found that vendors still lack adequate information pertaining to a healthy meal. This was observed when vendors explained their perception of a balanced meal where some perceived it to be the one having more than two kinds of foods regardless of the food groups, e.g., a meal consisting of stiff porridge, meat, and beans would be considered as balanced. In order to ensure accurate information, especially regarding a balanced diet, it is important for vendors to be introduced to and be familiarized with “My Plate Guidelines”, which gives a proper guide in ensuring meals prepared are adequate (Cohen et al., 2017). Safety of the food from proper preparation to clean utensils were also among the aspects considered by vendors. This is in contrast with the vendor's perspectives obtained in the study conducted in Nigeria, where vendors did not show concern on hygiene, cleanliness, and safety issues (Nordhagen et al., 2022). Although studied vendors were concerned, ensuring clean environment during meal preparation and meal serving is still a challenge due to a lack of appropriate facilities such as sewage and proper waste disposal systems, as well as lack of clean and safe water. The way foods are prepared and cooked is an essential step in ensuring the healthiness of a meal (Mills et al., 2017). This was considered by some vendors while others misinterpreted it with overcooking of foods, particularly vegetables. This finding suggests that vendors' perceptions are not primarily focused on the nutritional aspects of a healthy meal, such as the inclusion of the major five food groups (food variety) in the right proportions, moderation of salts, sugars, and fats/oils, and issues of calorie intake. Comparable findings were obtained by Hill et al. (2019) in South Africa where it was observed that vendors had a lack of nutrition knowledge regarding healthy diets.

Female vendors, vendors with primary and tertiary education, and experienced vendors had better perceptions of healthy meals. This is consistent with findings by Bärebring et al. (2020) where females had better health perceptions than their male counterparts. This is most likely due to women being more concerned with the healthiness of diets taken and thus being more aware of healthy meals (Perry et al., 2016). These results call for more efforts toward male vendors whose contribution to provision of street meals is not negligible.

Formal education was found to play a part in influencing better perceptions of healthy meals among the vendors. This is most likely due to their exposed knowledge of issues related to food and health. In schools, people get exposed to issues related to safe and healthy meals and particularly on how to ensure food safety and appropriate food preparations. Education can influence perception, as was found in the study by Williams et al. (2014) in which health perceptions of individuals improved after an education intervention was conducted. Thus, it is important to ensure that people engaging in food vending business were enrolled in secular education or have gone through trainings to ensure that people engaging in the business are knowledgeable.

Having more vending experience was shown to be associated with better perceptions, which might be due to frequent encounters with health officers who provide health and nutrition education during inspections. Other than that, experienced vendors with a greater customer base seek to understand the needs of their customers, some of whom prefer healthy meals. This exposure to health‐concerned consumers gives vendors an insight into the healthiness of meals they offer, thus influencing their perceptions. This is consistent with a study by Plasek et al. (2020), which found that meal providers preferred to consider their customers' needs and provide meals and food items that meet those needs.

### 4.2 Attitudes of street food vendors

Based on the results from this study, vendors have a generally positive attitude toward preparation of nutritious and healthy meals. The attitudes of the vendors were satisfactory, except for the healthiness of their own meals, as well as the importance of considering the energy content of the foods they offer, which were slightly lower. The attitudes of street food vendors regarding healthy eating have a significant impact on how well meals are prepared. Thus, there is a strong linkage between positive attitudes of street food vendors in preparing, offering, and maintaining healthy street meals (Tuglo et al., 2021). This could be an opportunity to intervene with street food vendors, who might be more willing to take part, for instance, in nutritional education efforts meant to change and adopt better meal preparations.

While there was a relatively lower positive attitude toward the healthiness of meals that vendors prepare, most vendors showed a strong positive attitude that unhealthy meals result in dietary‐related diseases, lots of fats/oil is bad for health, and that they should prepare healthy meals. This may be due to the increase in awareness of dietary‐related diseases and their potential risks to the general population (Alkerwi et al., 2015; Liao et al., 2019; Scalvedi et al., 2021). Nowadays, there are numerous ways for people to acquire and get dietary information. This awareness may have influenced attitudes of the vendors toward certain dietary aspects that concerns meal preparations. Adamski et al. (2020) reported an increase in the accessibility of nutritional information particularly through the use of the Internet. Additionally, vendors had particularly strong positive attitudes toward meal composition in terms of variety (with the inclusion of vegetables and fruits), safety, right portions, and benefits brought by the healthy meal. However, there are still nutritional issues that are either not fully addressed, or of which vendors are not aware of. This was highlighted by the lower positive attitudes toward the importance of considering energy content of the foods they serve. Calorie/energy content of food is an important nutritional aspect that needs to be considered when serving meals to people of different age groups and occupations (Institute of Medicine, 2011). Street food vendors that participated in this study seemed to be less troubled by this fact; therefore, efforts should be made during interventions to ensure maximum coverage of necessary nutritional aspects that are required in preparing and serving nutritious and healthy meals.

The findings of the current study showed that positive attitudes toward healthy meals and the healthiness of meals prepared and sold by street food vendors were significantly associated with participants' sex (females), education level (primary to tertiary education), and vending experience (more than 1 year). Relatively more female vendors had a positive attitude than male vendors. This finding aligns with the traditional social context, where females typically seek healthier meals than males (Oakes & Slotterback, 2001). It also urges any future interventions made to motivate the male vendors. It has been claimed that education is crucial in raising street food vendors' awareness of food nutrition and thereby improving their attitudes to healthy meals (WHO, 2015). In Vietnam (Huynh‐Van et al., 2022) and Bangladesh (Hossen et al., 2021), for example, significant effects of education level on food safety attitudes of food handlers were observed. Although marital status was not statistically significant, it has been identified that married individuals are often linked to more access to health information and resources (Sujarwoto et al., 2022). Previous studies have found a statistically significant association between marital status and vendor attitudes, with those married having a more positive attitude (Alhashim et al., 2022; Mukherjee et al., 2018). This is in agreement with the present study's findings, as those who were married had higher attitude scores. Street food vendors with greater vending experience displayed a more positive attitude than vendors with lower vending experience. Similar results were obtained in the study conducted by Abid et al. (2022). This could be attributed to the accumulated knowledge gained by experienced vendors from exposure to current nutritional issues related to food and food vending. This is encouraging, as it demonstrates that concern for healthy meals exists within the street food sector of the informal economy, but is still to a large extent market‐driven, as attitudes most likely relate to the desires and economic capacity of the consumers of street food.

## 5 CONCLUSION

This study concludes that the majority of vendors had good perceptions and positive attitudes toward healthy meals, although nutritional aspects of healthy meals were not of much concern. These findings imply that there is a chance for willingness of street food vendors to improve the quality of their meals. Thus, this study recommends the government through responsible ministries should ensure the provision of adequate nutrition and health education to vendors. Responsible authorities like Tanzania Food and Nutrition Center (TFNC) should formulate appropriate guidelines for the preparation of safe and healthier meals. Guidelines should have more emphasis on the provision of foods such as fruits and vegetables, fish, whole grains, legumes, and other healthier foods, and limiting the amount of fats/oils, salt, and use of red meat. Local governments, through nutrition officers, should cooperate with other stakeholders to ensure street food vendors are notified for various healthy meal trainings and they follow guidelines and legislations that are set. Nongovernmental organizations and other related stakeholders should communicate to influence changes in perception and attitude through healthy meal preparation programs, such as mobile health, and increase in media coverage. Also, the “Healthy meal seal strategy” can be used to approve vendors who use healthier ingredients, safe food handling, etc. This will motivate vendors to provide healthier meals. Future studies on nutritional knowledge and practices of vendors concerning healthy meals are worth conducting. Analysis of nutritional composition of the current street meals sold by the vendors should also be taken into consideration, which can be conducted based on the proposed protocol by WHO's “FEED cities” Project: A Comprehensive Characterization of the Street Food Environment in Cities. Project protocol. Copenhagen, Denmark: World Health Organization Regional Office for Europe, 2019 (WHO, 2019a).

### 5.1 Strengths and limitations

This was one of the few studies to explore perceptions and attitudes of street food vendors regarding the healthiness of meals sold in Urban Area – Tanzania. Thus, the findings may provide baseline information for public health practitioners to design and implement an evidence‐based intervention across the country to improve the quality of meals offered on the streets. Additionally, the study employed a population‐based sampling procedure that aptly represented the population of street food vendors’ perspectives and attitude in Tanzania in general and specifically in Dodoma city. The limitations of this study include the cross‐sectional nature of the study design, which does not allow establishment of cause and effects of the obtained information. Further studies that incorporate a longitudinal observational design should be looked into. This will provide more insights into the cause–effect relationship in regard to the perceptions and attitudes of street food vendors toward the healthiness of meals prepared and sold and their changes over time.

## AUTHOR CONTRIBUTIONS

**Zalia Omary Basheikh:** Conceptualization (equal); data curation (lead); formal analysis (lead); investigation (equal); methodology (equal); resources (lead); writing – original draft (lead); writing – review and editing (lead). **Theresia Jumbe:** Conceptualization (equal); data curation (supporting); methodology (equal); resources (supporting); supervision (lead); writing – original draft (supporting); writing – review and editing (supporting). **Kissa Kulwa:** Conceptualization (equal); data curation (supporting); methodology (equal); resources (supporting); supervision (lead); writing – original draft (supporting); writing – review and editing (supporting).

## FUNDING INFORMATION

This study was privately sponsored by the authors.

## CONFLICT OF INTEREST STATEMENT

The authors declare no conflict of interest.

## Data Availability

The data that support the findings of this study are available on request from the corresponding author. The data are not publicly available due to privacy or ethical restrictions.
